# Functions of Cancer-Derived Extracellular Vesicles in Immunosuppression

**DOI:** 10.1007/s00005-016-0453-3

**Published:** 2017-01-18

**Authors:** Liliana Czernek, Markus Düchler

**Affiliations:** Department of Bioorganic Chemistry, Centre of Molecular and Macromolecular Studies, Polish Academy of Sciences, Sienkiewicza 112, 90-363 Lodz, Poland

**Keywords:** Exosomes, Extracellular vesicles, Cancer immunosuppression, Suppressor cells, Immune escape

## Abstract

Extracellular vesicles, including exosomes, constitute an important element of intercellular communication by carrying a variety of molecules from producer to target cells. The transport of mRNA and miRNA can directly modulate gene expression in the target cells. The miRNA content in exosomes is characteristic for the cell from which the vesicles were derived enabling the usage of exosomes as biomarkers for the diagnosis various diseases, including cancer. Cancer-derived exosomes support the survival and progression of tumors in many ways and also contribute to the neutralization of the anti-cancer immune response. Exosomes participate in all known mechanisms by which cancer evades the immune system. They influence the differentiation and activation of immune suppressor cells, they modulate antigen presentation, and are able to induce T-cell apoptosis. Although cancer-derived exosomes mainly suppress the immune system and facilitate tumor progression, they are also important sources of tumor antigens with potential clinical application in stimulating immune responses. This review summarizes how exosomes assist cancer to escape immune recognition and to acquire control over the immune system.

## Introduction

In the early 1980s, two research groups described that the transferrin receptor of sheep reticulocytes was secreted via little-known vesicular forms (Pan and Johnstone 1984). The same researchers described the mechanism of small vesicle secretion showing that the release of membrane vesicles was preceded by inward budding of an intracellular endosome forming a multi-vesicular body (MVB), which could then fuse with the plasma membrane (Pan et al. 1985). Rose Johnstone used the term “exosomes” for the first time to describe small membrane vesicles formed in MVBs (Johnstone et al. 1987). The original function attributed to membrane vesicles was the removal of cell debris. The thinking about membrane vesicles as “trash cans” of the cell was derived from the knowledge about the role of lysosomes as degradation centers (Luzio et al. 2007). Since the finding that exosomes can modulate the immune system, extracellular vesicles gained growing interest (Raposo et al. 1996). The enthusiasm was further increased after the discovery of mRNA and miRNA inside exosomes (Valadi et al. 2007). These studies opened the door to the new research field of exosome functions in intercellular communication, their biomarkers, and their potential role as therapeutic tools.

## Classification of Extracellular Vesicles

Cells release different kinds of extracellular vesicles (EVs) of varying sizes and biogenesis. Their classification distinguishes three main subpopulations/classes based on the vesicle’s origin. The smallest vesicles are of endocytic origin, exosomes, with 40–150 nm in diameter (Baietti et al. 2012; Colombo et al. 2013). Ectosomes, also called shedding microvesicles, with a diameter of 100–1000 nm are produced by outward protrusion or budding from the plasma membrane (Muralidharan-Chari et al. 2009; Théry et al. 2009). The most heterogeneous group of vesicles ranging from 50 up to 5000 nm in diameter is apoptotic bodies. Their biogenesis is based on fragmentation of apoptotic cells during programmed cell death (Mathivanan et al. 2010; Théry et al. 2009). A common feature of all vesicle classes is their membrane structure, a lipid bilayer with the same topological orientation as the plasma membrane (Trajkovic et al. 2008). Although the origin of microvesicles and exosomes is well known, the experimental discrimination of these vesicles types is difficult, and so the terms are sometimes subsumed as extracellular vesicles. In this review, we follow the terminology used by the authors.

## Exosome Biogenesis

The process of exosome biogenesis is not fully understood. It starts within endosomes which are responsible for regulated trafficking of proteins and lipids between subcellular compartments of the secretory and endocytic pathway (Lemmon and Traub 2000). The cargo of endosomes can enter recycling circuits to return membrane components back to the plasma membrane, or can be sorted into lysosomes for degradation (Huotari and Helenius 2011). The content of cholesterol is associated with the fate of MVBs; cholesterol-poor MVBs are appointed for lysosome fusion and degradation (Möbius et al. 2002). Exosomes formed within MVBs are released via exocytosis into the extracellular space when cholesterol-rich MVBs fuse with the plasma membrane (Kalra et al. 2012).

During vesicle formation, cellular components, extracellular ligands, and other endocytosed molecules, such as receptors, are packed into the vesicles (Gould and Lippincott-Schwartz 2009). Molecules from the early endosomes, such as the tetraspanin CD63, or LAMP1 and LAMP2, are released through the vesicles (Colombo et al. 2014; Jaiswal et al. 2002; Raposo et al. 1996). The ESCRT (endosomal sorting complex required for transport) machinery is involved in the budding process, as well as in the controlled sorting of proteins into exosomes. The ESCRT machinery consists of four complexes, ESCRT-0 (Hrs), ESCRT-I (TSG101 and Vps28), ESCRT-II (Vps22), and ESCRT-III (Alix and Vps2), which sort ubiquitinylated proteins to the late endosomes. The ESCRT-III complex was shown to promote intraluminal budding of vesicles in endosomes which results in maturation of the cargo-containing vesicles (Colombo et al. 2013; Kowal et al. 2014). An ESCRT-independent packaging mechanism was also proposed involving glycolipoprotein microdomains (lipid rafts) (Trajkovic et al. 2008). Besides a big range of proteins, also nucleic acids like mRNA, miRNA, or DNA can be found in exosomes (http://www.exocarta.org) (Thakur et al. 2014; Valadi et al. 2007). Interestingly, the miRNA contents of exosomes do not entirely parallel the miRNA composition inside the cell indicating selective loading mechanisms (Rappa et al. 2013). For the selection of miRNA for exosomal export, several potential routes were described, one depending on neural sphingomyelinase 2 (Kosaka et al. 2013), a second based on uridylation versus adenylation of the 3′end of the miRNAs (Koppers-Lalic et al. 2014), a third one involving sumoylated heterogeneous nuclear ribonuleoprotein (hnRNPA2B1) binding to a GGAG motif in the 3′part of miRNA sequences to be packed into exosomes (Villarroya-Beltri et al. 2013), and another one related to the RISC pathway (Gibbings et al. 2009). For the selective loading of mRNA into microvesicles, a 25-nucleotide sequence motif in the 3′-UTR of exported mRNAs was described (Bolukbasi et al. 2012). In cancer cell-derived exosomes, also fragments of chromosomal DNA were identified (Kahlert et al. 2014), their sorting mechanism into the vesicles has not yet been defined. The analysis of the miRNA content of exosomes allows to draw conclusions about the cell type from which the exosomes originated. Thus, determining the miRNA profile in extracellular vesicles derived from bodily fluids of diseased persons has a huge potential for diagnostic purposes (Miller and Grunewald 2015; Verma et al. 2015).

## Cancer’s Immune Escape

The immune system provides a defense against attacks of foreign invaders, such as bacteria, viruses, and parasites, or the growth of cancer cells. Once it recognizes non-self antigens, it activates multiple chemical and physiological processes constituting the immune response (Kindt et al. 2007). The immune response comprises innate and adaptive immunity. The components of the innate response include antigen-presenting cells (APCs) like macrophages or dendritic cells (DCs) that are responsible for phagocytosis, digestion, and presentation of pathogen-derived antigens on the cell surface, and natural killer (NK) cells that directly destroy infected or transformed cells. The innate immune response is followed by the adaptive one which is based on activation of specific B and T lymphocytes. T cells are highly specialized cells that not only coordinate (T-helper: Th) or suppress (T-regulatory: Treg) the immune response, but also destroy infected cells (T-cytotoxic: CTL). B cells secrete antibodies which mark infected cells or pathogens to promote their elimination from the organism. The T-cell and B-cell responses include the production of memory cells against the pathogen enabling quicker immune response in future challenges (Kindt et al. 2007).

Cancer cells have to express antigens which are recognized as non-self to elicit an immune response. Such tumor-associated antigens (TAA) are either mutated cellular proteins, or molecules with differences in posttranslational modifications (Finn 2012). TAA-derived peptides produced by the proteasome are presented through major histocompatibility complex (MHC) I complexes on the cell surface and recognized by CTLs resulting in tumor cell killing. The strategies used by tumors to escape this destruction include the impairment of the executory capacity of the immune system, and hiding from recognition by immune cells through the loss of target antigen expression. Defective antigen presentation can be caused by the down-regulation of the antigen processing machinery which may affect the MHC-I pathway and other involved proteins like the proteasome subunits LMP2 (latent membrane protein 2) and LMP7, the transporter associated with antigen processing, and tapasin (Garrido et al. 1997; Hicklin et al. 1999; Johnsen et al. 1999; Restifo et al. 1993; Rotem-Yehudar et al. 1996). When the expression of TAA is down-regulated, CTL no longer recognize the tumor cells (Maeurer et al. 1996). About 20 years ago, mutations in the β2-microglobulin gene have been identified in metastatic melanoma cells resulting in the absence of HLA class I antigens on the cell surface (Benitez et al. 1998).

The production of immune suppressive cytokines by cancer cells or non-cancer cells in the tumor microenvironment exerts a powerful suppression of the anti-cancer immune response. Among these cytokines are transforming growth factor (TGF)-β, tumor necrosis factor (TNF)-α, interleukin (IL)-1, IL-6, IL-8, IL-10, and type I interferons (IFNs) (Pasche 2001; Lind et al. 2004; Matsuda et al. 1994). Furthermore, vascular endothelial growth factor (VEGF) has the ability to suppress proper T-cell development and function (Ohm et al. 2003). TGF-β and IL-10 can shift the balance from a Th1 response executed by cytotoxic T cells towards an antibody-based Th2 response (immune deviation) (Maeda and Shiraishi 1996). Induction of immune tolerance may also occur through down-regulation of co-stimulatory molecules on APCs. Engagement of the T-cell receptor (TCR) in the absence of co-stimulation induces anergy or tolerance in T cells (Staveley-O’Carrol et al. 1998). Tumors even eliminate tumor-specific CTLs by expressing ligands to death receptors which trigger T-cell apoptosis (Bogen 1996). Advanced cancer-induced immunosuppression results in the induction and activation of immune suppressor cells like myeloid-derived suppressor cells (MDSCs) and Treg cells. Treg cells generally suppress the activity and proliferation of effector T cells (Shevach 2002), fulfil an important function to maintain immune tolerance to self-antigen, and are critical in the suppression of autoimmune diseases. It was shown that tumor-derived Tregs have comparatively higher suppressive activity than naturally occurring Tregs (Yokokaw et al. 2008; Gasparoto et al. 2010). The induction and activation of cancer-antigen-specific Treg cells seem to be the major mechanism of tumor immune escape (Vinay et al. 2015).

## Specific Features of Cancer Cell-Derived Extracellular Vesicles

Cancer cells release increased amounts of exosomes compared to their non-transformed counterparts (Pap et al. 2011). Chemotherapy or photo-dynamic treatment further boosts the release of extracellular vesicles (Aubertin et al. 2016). Cancer-derived exosomes were shown to contribute to tumor angiogenesis, to transport growth promoting proteins, such as mutant KRAS, epidermal growth factor receptor (EGFR), and SRC family kinases, to induce therapy resistance by removal of chemotherapeutic drugs, and to prepare metastatic niches for the colonization of circulating cancer cells (reviewed in Miller and Grunewald 2015). For example, Al-Nedawi et al. (2008, 2009) showed that exosomes transferred functional EGFR from cancer cells to endothelial cells. Subsequently, VEGF secretion was induced which triggered autocrine VEGF signaling by binding to the endothelial VEGFR-2 and resulted in neovascularization.

Due to their complex structure, exosomes may contribute to both, stimulation and suppression of immune responses. Whether cancer-derived exosomes stimulate immunity or tolerance seems to also depend on the amount of transferred vesicles with a suppressive effect at high vesicle concentration (Hellwinkel et al. 2016).

## Immune Stimulation

Exosomes can promote immune responses by regulating signals for both, adaptive and innate immune responses (Zhang et al. 2014). Cancer exosomes bear MHC class I and class II complexes at their surface and were able to function as antigen-presenting vesicles to directly activate T cells (Raposo et al. 1996). Exosomes derived from both human and murine B lymphocytes induced antigen-specific MHC class II-restricted T-cell responses. Primed antigen-specific T cells were efficiently stimulated by MHC II complexes on exosomes secreted from activated B cells, suggesting a role for B-cell-derived exosomes to modulate an ongoing immune response or to maintain antigen-specific memory T cells. However, T-cell priming was necessary, as B-cell-derived exosomes could stimulate primed CD4^+^ T cells, but not naïve T cells (Muntasell et al. 2007; Raposo et al. 1996).

In an indirect, but more efficient pathway of immunostimulation, exosomes transfer tumor antigens to DCs and other APCs (Denzer et al. 2000; Morelli et al. 2004; Wolfers et al. 2001). Tumor antigens from cancer-derived exosomes are captured and presented by APCs to induce efficient anti-tumor immune responses (Rao et al. 2016). This pathway has extensively been exploited for cancer vaccination and immunization with exosomes isolated from ascites in colorectal cancer patients who were explored in phase I clinical trials (Dai et al. 2008). Human DCs loaded with glioma-derived exosomes activated a tumor-specific CTL response in vivo (Bu et al. 2011). Exosome treatment stimulated the up-regulation of MHC II molecules and the co-stimulatory receptors CD80 and CD86 on the DCs. The efficiency of anti-tumor immunity induction could be increased by either stimulation of Rab27a over-expression to boost exosome secretion (Li et al. 2013), or by expressing cytokines, such as TNF-α, in the exosome producing cells (Xie et al. 2010). DCs loaded with tumor-derived exosomes were more efficient in vaccination of mice than DCs loaded with tumor cell lysates (Gu et al. 2015; Yao et al. 2013). Furthermore, targeting of tumor antigens to exosomes improved the vaccination efficacy (Rountree et al. 2011). Another successful vaccination approach employed exosomes loaded with tumor peptide antigen and α-galactosylceramide to specifically target and activate NKT cells (Gehrmann et al. 2013). Increased levels of heat-shock proteins on exosomes derived from heat-shocked lymphoma cells improved the anti-tumor immune response (Chen et al. 2006). The presentation of tumor antigens to DCs by exosomes could also be improved by modification of exosomes with immunostimulatory CpG DNA (Morishita et al. 2016).

Dendritic cells not only take up, but also produce vesicles to transfer MHC/peptide complexes to other immune cells (André et al. 2004). Such exosomes derived from TAA-loaded DCs were directly used as cancer vaccines (Escudier et al. 2005; Mahaweni et al. 2013; Näslund et al. 2013; Viaud et al. 2010). Théry et al. (2002) showed that DC-derived exosomes could stimulate naïve CD4^+^ T cells in vivo. Incorporation of poly(I:C), a ligand for Toll-like receptor 3 (TLR3), into antigen-loaded exosomes was demonstrated to improve the vaccination efficiency of DC-derived vesicles (Damo et al. 2015). This approach could be further improved using exosomes derived from mature DCs pulsed with DC-derived exosomes (Hao et al. 2007). The usage of DC-derived exosomes for cancer vaccination has reached phase I clinical trials which demonstrated the safety of this approach (Escudier et al. 2005).

Pro-inflammatory effects of tumor-derived exosomes were described for macrophages. Exosomes derived from melanoma cells affected the cytokine and chemokine profile in macrophages (Marton et al. 2012). Wu et al. (2016) demonstrated that macrophages activated by gastric cancer-derived exosomes acquired a pro-inflammatory phenotype. Exosome uptake by macrophages stimulated the NF-κB pathway to increase the expression of pro-inflammatory factors, such as IL-6 and TNF-α (Wu et al. 2016). A similar NF-κB-dependent up-regulation of inflammatory factors via TLR2 was found by Chow et al. (2014). Interestingly, in this study, the inflammatory response was elicited only by exosomes secreted by breast cancer cells but not by vesicles from non-cancerous cell lines.

In conclusion, exosomes derived from cancer cells or TAA-loaded APCs provide a promising tool for cancer immunotherapy and vaccination due to their immunogenicity which can be further increased by innovative approaches.

## The Functions of Cancer-Derived Exosomes in Immunosuppression

**Fig. 1:**
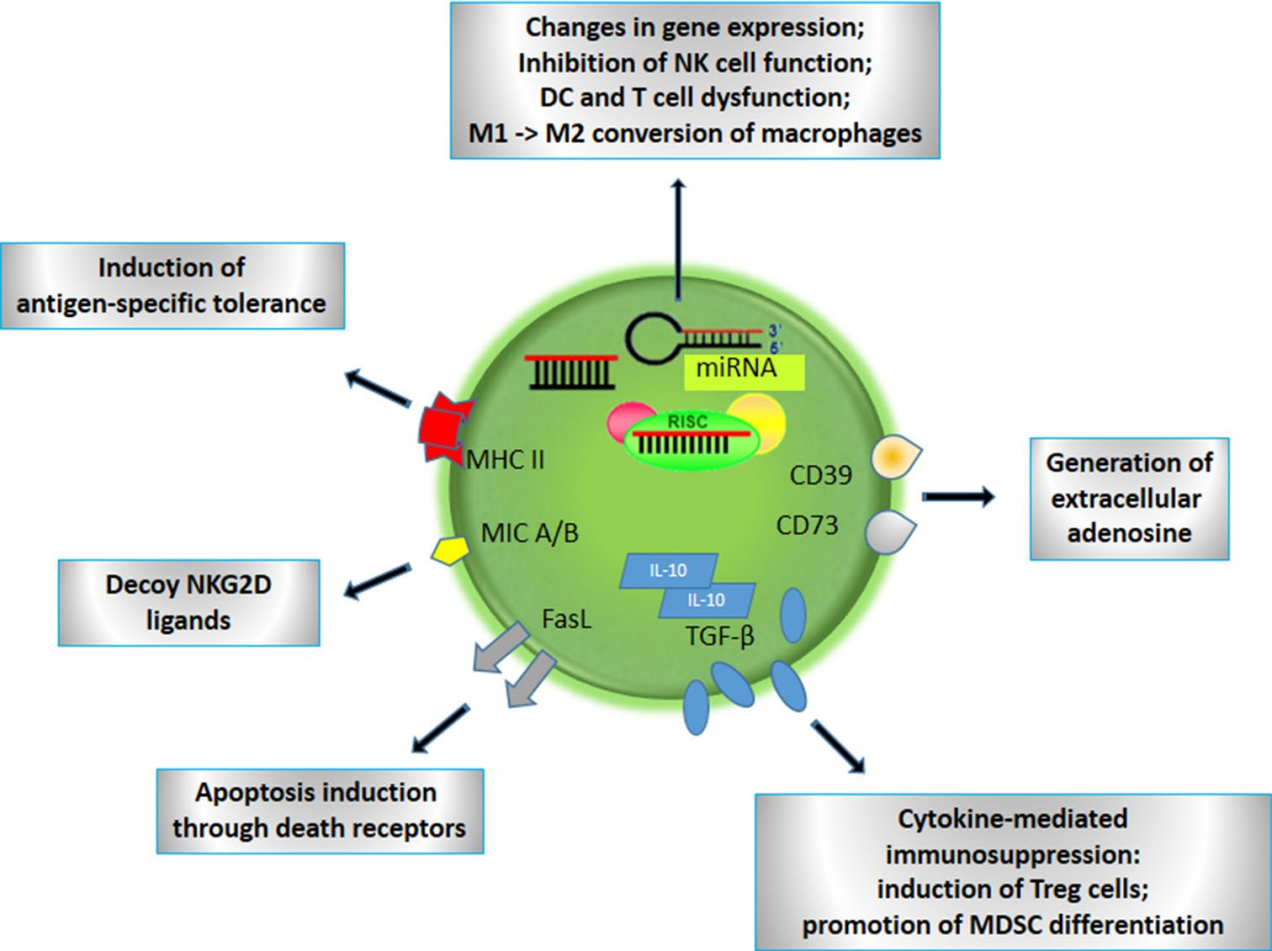
Schematic representation of the most important functions of cancer-derived exosomes in immunosuppression. Active molecules transported by exosomes and their effects on immune cells are indicated

To escape destruction by the immune response, tumors avoid to be recognized by cytotoxic cells, directly impair the functioning of APCs or cytotoxic cells, or induce suppressor cells which consequently shut down immune reactions. Immune cells are even converted into supporters of tumor growth and survival. Exosomes participate in all these strategies through proteins exposed at their surface, and intra-vesicular cytokines and nucleic acids (Fig. 1).

### Defective Antigen Presentation

The body’s own cells are protected from the attack of cytotoxic T cells by exposing on their surface MHC class I molecules presenting peptides derived from un-mutated normal proteins. Tumor cells expressing MHC-I/TAA-peptide complexes instead are destroyed by cytotoxic T cells. To escape this destruction, cancer cells may down-regulate MHC-I expression. However, according to the “missing self” hypothesis, cells lacking MHC-I/self-peptide expression (“missing self”) are recognized and destroyed by NK cells (Ljunggren and Kärre 1990). In this way, the immune system counteracts the escape strategy of transformed and virus infected cells through down-regulation of MHC-I. To avoid destruction by NK cells after shutting down MHC-I expression, cancer cells have to find a way to inhibit NK-cell cytotoxicity. One possibility is the shedding of exosomes which affect the cytotoxic ability of NK cells (Clayton et al. 2008). NK-cell activity is regulated by the interplay of activating and inhibitory receptors. One of the activating receptors is NKG2D (NK group 2, member D) which interacts with its human ligands MIC-A and MIC-B (MHC class I chain-related proteins A and B) and ULBP (UL-16-binding protein) (Groh et al. 2002; Raulet and Guerra 2009). Hedlund et al. (2011) showed that NKG2D ligands (MIC-A/B and ULBP 1 and 2) are expressed and secreted on exosomes. The authors demonstrated that NKG2D ligand-carrying exosomes impair NKG2D-mediated NK-cell cytotoxicity by acting as a decoy and, thus, contribute to the immune evasion of leukemia/lymphoma cells (Hedlund et al. 2011). Exosomes exposing NKG2D ligands are further able to down-regulate NKG2D expression on NK cells. This was shown for exosomes produced by human prostate cancer cells (Lundholm et al. 2014) and by acute myeloid leukemia blasts (Hong et al. 2014).

### Suppression of APCs and Cytotoxic T Cells

Exosomes carry a spectrum of membrane-bound factors which have been shown to mediate immune suppression, representing another mechanism utilized by tumors to evade anti-tumor functions of immune cells (Schuler et al. 2014). The prime target of direct immunosuppression is the cytotoxic T cell. Growth inhibition of CD8^+^ cytotoxic T cells mediated by glioblastoma derived exosomes was shown to promote tumor growth in mice (Liu et al. 2013). A similar observation was made with microvesicles isolated from the sera of head and neck cancer and melanoma patients, which impaired signaling and proliferation of CD8^+^ CTLs (Wieckowski et al. 2009).

#### Vesicle-Associated Immunosuppressive Cytokines

One of the major immunosuppressive cytokines is TGF-β which can be associated with and exposed at the exosome surface. The potency of this vesicular form to influence cell differentiation exceeds that of the soluble form (Webber et al. 2015). In acute myeloid leukemia, NK cells became suppressed by tumor-derived microvesicles via TGF-β1 on the exosome surface (Szczepanski et al. 2011), and breast cancer-derived exosomes suppressed T-cell proliferation through this cytokine (Rong et al. 2016).

#### Apoptosis Induction

Tumor-derived microvesicles were shown to induce T-cell apoptosis through the receptor-mediated pathway (Taylor et al. 2003; Wieckowski et al. 2009). For example, Fas ligand (FasL)-containing microvesicles from melanoma cells triggered apoptosis of Jurkat and other lymphoid cells. Engagement of the death receptor Fas by FasL resulted in apoptotic cell death, mediated by caspase activation (Andreola et al. 2002). Exosome-like particles derived from human colorectal cancer cells expressed FasL and TNF-α and triggered T-cell apoptosis in vitro and in vivo (Huber et al. 2005). In addition, exosomes produced by prostate cancer cells or human B-cell-derived lymphoblastoid cell lines suppressed T-cell responses through FasL-mediated induction of apoptosis (Abusamra et al. 2005; Klinker et al. 2014). Another pathway of apoptosis induction in CD4^+^ Th1 cells was described for exosomes derived from nasopharyngeal carcinoma cell. These exosomes carried high amounts of galectin-9 which triggered cell death by binding to its cellular receptor, Tim-3 (Klibi et al. 2009).

#### Gene Regulatory Effects

In general, exosomes can modify the transcriptional profile of the recipient cells by receptor activation, or by directly changing gene expression through delivered nucleic acids (Skog et al. 2008; Valadi et al. 2007). In a recent study, Muller et al. (2016) showed that signals delivered by cancer exosomes induced changes in the transcriptional profile of T cells and that immune response-regulating genes were preferentially targeted in T lymphocytes, especially in activated T lymphocytes. Cancer exosomes co-incubated with human CD4^+^ CD39^+^ Treg cells, conventional CD4^+^ T cells, or CD8^+^ T lymphocytes differentially regulated the expression of key immune function-related genes. The changes in mRNA expression levels were dependent on the cell type and the activation status. Incubation with cancer-derived exosomes increased the levels of critical immune inhibitory proteins, such as TGF-β, IL-10, COX-2, CD39, and CD73 (Muller et al. 2016).

While a role of exosomal mRNAs in cancer-mediated immunosuppression was not yet described, a few publications found an influence of exosome transported miRNAs. Ding et al. (2015) found increased levels of nine miRNAs in DCs treated with exosomes isolated from pancreatic cancer cells. Consequently, more than 200 mRNAs were down-regulated. They further demonstrated that miR-212 caused a decrease in MHC II expression by targeting regulatory factor X-associated protein, an important transcription factor for MHC II. In exosomes from nasopharyngeal carcinoma cells, five over-expressed miRNAs (hsa-miR-24-3p, hsa-miR-891a, hsa-miR-106a-5p, hsa-miR-20a-5p, and hsa-miR-1908) were identified which reduced MAP-kinase signaling in T cells thus altering proliferation and differentiation behavior (Ye et al. 2014). Recently, it was shown that hypoxia changed the immunosuppressive potential of extracellular vesicles. Microvesicles isolated from hypoxic lung carcinoma cells showed a stronger inhibition of NK-cell function than those isolated in normoxic conditions. The immunosuppressive effect was mediated by miR-23a, in addition to TGF-β (Berchem et al. 2015). MiR-4498 showed higher levels in hypoxic exosomes isolated from melanoma cells (own unpublished results) and might influence immune responses by targeting CD83, an immunostimulatory molecule critical for the activation of T cells (Su et al. 2016). In murine tumor models, mir-494 was shown to regulate the activity of MDSC (myeloid-derived suppressor cells), a major type of immunosuppressive cells (Liu et al. 2012).

#### Other Exosome-Triggered Direct Immunosuppressive Mechanisms

Additional mediators involved in immune suppression include CD39 and CD73 present on the surface of cancer-derived exosomes (Schuler et al. 2014; Smyth et al. 2013). CD39 and CD73 initiate an ectonucleotidase cascade that generates extracellular adenosine, which has suppressive effects on T cells. It is known that adenosine in the extracellular environment is a potent immune regulatory factor protecting cells and tissues from excessive immune-mediated damage and negatively regulates local immune responses. Exosomes secreted by cancer cells contributed to extracellular adenosine production and hence indirectly modulated immune effector cells (Clayton et al. 2011). An entirely different mechanism was described for melanoma-derived exosomes which raised ROS levels in T cells resulting in impaired TCR signaling due to zeta-chain inactivation (Söderberg et al. 2007).

Tumor exosomes also exerted a direct influence on mesenchymal stem cells (MSCs). MSCs are multipotent stromal cells with important function in tissue regeneration. MSCs support cancer progression and may create a local immunosuppressive microenvironment. Lung tumor cell A549-derived exosomes induced a pro-inflammatory phenotype of MSCs. Hsp70 on the surface of the exosomes triggered signaling through TLR2 leading to activation of NF-κB and elevated secretion of IL-6, IL-8 and monocyte chemotactic protein 1 by MSCs (Li et al. 2016). A summary of immunosuppressive effects elicited by cancer-derived exosomes is presented in Table 1.

**Table 1:** Summary of immunosuppressive effects elicited by exosomes

Source of exosomes	Molecule	Effect on immune cells	References
Jurkat and Raji cell lines	NKG2D ligands	Decoy for NKG2D receptor function	Hedlund et al. (2011)
Mesothelioma and various cancer cell lines	TGF-β	NKG2D down-modulation	Clayton et al. (2008)
Head and neck squamous cell carcinoma; melanoma cell lines	FasL	Promotion of Treg cell expansion and the demise of anti-tumor CD8^+^ effector T cells, induction of TGF-β production by Treg	Wieckowski et al. (2009)
Colorectal cancer	FasL, TNF-α	CD8^+^ T-cell apoptosis	Abusamra et al. (2005)
Ovarian cancer	FasL	Apoptosis and caspase-3 activation within T cells	Taylor et al. (2003)
Melanoma	FasL	Apoptosis in lymphoid cells	Andreola et al. (2002)
Colorectal cancer	FasL, TNF-α	T-cell apoptosis	Huber et al. (2005)
B-cell lymphoma	FasL	T-cell apoptosis	Klinker et al. (2014)
EBV-associated NPC	Galectin-9	Apoptosis in EBV-specific CD4^+^ cells	Klibi et al. (2009)
Acute myeloid leukemia	Membrane-associated TGF-β	Suppression of NK-cell function	Szczepanski et al. (2011)
Various cancer cell lines	CD39 and CD73	Generation of extracellular adenosine	Clayton et al. (2011)
Pancreatic cancer	Nd	Increased levels of 9 miRNAs, down-regulation of >200 mRNAs	Ding et al. (2015)
Lung carcinoma	miR-23a TGF-β	Inhibition of NK-cell function	Berchem et al. (2015)
Melanoma	Nd	TCR zeta-chain inactivation through ROS	Söderberg et al. (2007)
Head and neck cancer cell line	Nd	Regulation of immune response-related genes in T cells, up-regulation of TGF-β, IL-10, COX-2, CD39, CD73 and adenosine production	Muller et al. (2016)
Pancreatic cancer	miR-203	Down-regulation of TLR4 and downstream cytokines in DCs	Zhou et al. (2014)
Nasopharyngeal carcinoma	miR-24-3p, miR-891a, miR-106a-5p, miR-20a-5p, miR-1908	T-cell dysfunction through down-regulation of the MAPK1 and JAK/STAT pathways	Ye et al. (2014)
Lewis lung carcinoma cell line and human embryonic kidney cell line	miR-214	Down-regulation of PTEN and promotion of Treg expansion	Yin et al. (2014)
Mesothelioma and various cancer cell lines	TGF-β	Induction of human Treg cells	Clayton et al. (2007)
Nasopharyngeal carcinoma	Nd	Conversion of the conventional T cells into Treg	Mrizak et al. (2014) Ye et al. (2014)
Colorectal cancer	TGF-β	Induction of Treg cells	Yamada et al. (2016)
Melanoma	Nd	Generation of CD14^+^HLA-DR^−/low^ cells secreting TGF-β	Valenti et al. (2006)
Murine mammary adenocarcinoma	Nd	Blockage of myeloid precursor differentiation into DCs	Yu et al. (2007)
B16 mouse model for human melanoma	Nd	MDSC inducion involving MyD88	Liu et al. (2010)
Mammary carcinoma	TGF-β, PgE2	Promotion of MDSC differentiation	Xiang et al. (2009)
Multiple myeloma	Nd	Promotion of MDSC viability and proliferation	Wang et al. (2016)
Renal cancer	Hsp70	TLR2 mediated Stat3 activation in MDSC	Diao et al. (2015) Xiang et al. (2010)
Various cancer cell lines	Hsp72	Stat3 activation and IL-6 production in MDSC	Chalmin et al. (2010)
Murine thymoma	Nd	Induction of B cells with inhibitory function	Yang et al. (2012a)
Esophageal cancer	Nd	Induction of regulatory B cells expressing TGF-β	Li et al. (2015)
Ovarian cancer	miR-222	Conversion of M1 macrophages into the M2 phenotype	Ying et al. (2016)

*Nd* not defined, *EBV* Epstein–Barr virus, *NPC* nasopharyngeal carcinoma, *ROS* reactive oxygen species, *PTEN* phosphatase and tensin homolog

### Induction and Activation of Immunosuppressive Cells

Tumor-derived exosomes were found to direct the differentiation of naïve immune cells towards an immunosuppressive phenotype and to activate the suppressor cells. The generation, expansion, and activation of Treg cells can be driven by cancer-derived exosomes (Szajnik et al. 2010; Wieckowski et al. 2009). Clayton et al. investigated that whether tumor-derived exosomes could modify lymphocyte IL-2 responses. Mesothelioma-derived exosomes induced human Treg cells (CD4^+^CD25^+^Foxp3^+^) which exerted dominant anti-proliferative effects on other T and NK lymphocytes in response to IL-2. Due to an exosome-related mechanism, IL-2 responsiveness was shifted in favor of Treg cells and away from cytotoxic cells (Clayton et al. 2007). Exosomes from nasopharyngeal carcinoma recruited Treg cells into the tumor through the chemokine CCL20, and mediated the conversion of the conventional T cells into Treg cells (Mrizak et al. 2014).

Under the influence of exosomes secreted by nasopharyngeal carcinoma cells, T-cell proliferation was inhibited, while Treg induction was stimulated (Ye et al. 2014). Furthermore, the production of IL-2, IL-17, and IFN-γ was decreased indicating impaired immune stimulation. Extracellular vesicles from colorectal cancer cells activated Smad signaling in T cells through exosomal TGF-β1 changing the phenotype into Treg-like cells (Yamada et al. 2016). In addition, miRNAs transported via microvesicles participated in the induction of the Treg cell phenotype, as shown for MiR-214 which mediated reduction of the PTEN (phosphatase and tensin homolog) level in mouse peripheral CD4^+^ T cells (Yin et al. 2014). Interestingly, exosomes were described to elicit antigen-specific immunosuppression (Yang et al. 2011, 2012b). The application of tumor-derived exosomes suppressed a delayed-type hypersensitivity response to a model antigen in an antigen-specific manner. The exact mechanism is not known but might include modulation of APCs.

Tumor-derived vesicles are able to impair DC development and to induce MDSCs (Valenti et al. 2006). The presence of cancer exosomes severely impaired the differentiation of DCs from murine bone marrow precursors or from human monocytes (Yu et al. 2007). The induction of IL-6 expression in the precursor cells was partially responsible for the observed block in DC differentiation. Valenti et al. (2006) showed that tumor-derived vesicles not only inhibited DC differentiation, but actively skewed precursors toward the acquisition of a MDSC phenotype. These cells mediated negative regulation of effector cells, e.g., through the secretion of soluble TGF-β (Valenti et al. 2006). Exosomes derived from murine breast carcinomas triggered the MDSC differentiation pathway, and this activity was dependent on prostaglandin E2 (PgE2) and TGF-β (Xiang et al. 2009). In addition, exosomes released by human multiple myeloma cells promoted the viability and proliferation of MDSCs (Wang et al. 2016). MDSC survival was supported by the activation of Stat3 (Wang et al. 2015). Renal cancer cell-derived exosomes induced the phosphorylation of Stat3 in MDSCs in a TLR2-dependent manner through the transfer of heat-shock protein 70 (Hsp70) (Diao et al. 2015). Blocking the Hsp70/TLR2 interaction with a peptide aptamer reduced the ability of tumor-derived exosomes to stimulate MDSC activation (Gobbo et al. 2015). The dependence of MDSC expansion on TLR2 was further investigated and confirmed by Xiang et al. (2010). In addition, membrane-bound Hsp72 in exosomes derived from human and murine cancer cell lines activated MDSCs and stimulated their suppressive function via Stat3 activation and IL-6 production (Chalmin et al. 2010). The involvement of MyD88 in the recruitment and activity of MDSC after exposure of bone marrow derived cells to tumor exosomes was shown in mice (Liu et al. 2010). MyD88 is a downstream effector of TLR signaling, and thus the findings corroborate the critical involvement of the TLR pathway.

In addition, the promotion of B cells with inhibitory activity by cancer exosomes was reported (Yang et al. 2012a). Mycoplasma-infected murine thymoma and melanoma cells released exosomes that induced IL-10 production in splenic B cells. Another study described how under the influence of esophageal cancer-derived microvesicles naïve B cells developed into immunosuppressive regulatory B cells expressing TGF-β (Li et al. 2015).

The conversion of cancer-suppressive cells into supporters of tumor growth and survival by exosomes was described for macrophages. Macrophages are the most abundant immune cells within the tumor microenvironment. Macrophages can be polarized into a cancer-suppressive M1 or a tumor supportive M2 phenotype. Exosomes from epithelial ovarian cancer were shown to shift macrophages towards the M2 phenotype (Ying et al. 2016). The involvement of miR-222 transferred by the exosomes was proposed through down-regulation of SOCS3. A similar activity was attributed to miR-494 that inhibited macrophage polarization and switched them towards the immunosuppressive M2 type (Zhao et al. 2016). In a co-culture system of murine cell lines, pancreatic cancer cell-derived exosomes shifted macrophage polarization to the M2 phenotype (Su et al. 2016). Over-expression of miR-155 and miR-125b-2 in the cancer cells reverted this effect and resulted in M1 polarized macrophages upon exosome exposure. The differentiation of monocytes into macrophages in the presence of colon cancer cell-derived EVs revealed increased IL-10 secretion and a mixed M1/M2 polarization status which, after longer incubation time, switched to the regulatory M2 phenotype (Baj-Krzyworzeka et al. 2016).

The great variety of mechanisms to induce immunosuppressive cells exemplifies the potential of EVs to modulate the function of recipient cells by the transfer of bioactive molecules.

### Exosomes Derived from Cancer Cells and Normal Cells Share Immune Signaling Functions

Recently, a very comprehensive review of the physiological roles of EVs was published covering their currently known functions in healthy organisms (Yáñez-Mó et al. 2015). This overview confirmed the crucial importance of EVs in intercellular signal transduction with effects on coagulation and angiogenesis, reproduction, embryonic development, tissue repair, organ homeostasis, and immunity. Communication between immune cells is one of the best characterized roles of exosomes and other EVs. Increased release of exosomes was observed upon interaction of DCs or B cells with T cells (Buschow et al. 2009; Muntasell et al. 2007), or when T-cell antigen receptors were engaged (Blanchard et al. 2002).

EVs from different sources exert immunosuppressive effects. Exosomes released from CD4-positive Th cells could suppress the activity of cytotoxic T cells (Zhang et al. 2011). Tolerogenic EVs derived from non-malignant cells contribute to the establishment and maintenance of the immune-privileged status of certain tissues. An important example is the human embryo which is protected during pregnancy from immune attacks by the exchange of EVs at the interface between the maternal placenta and the fetus. Placenta-derived exosomes were shown to suppress the immune system by carrying NKG2D ligands (MIC and the ULBP), which bind and down-regulate the NKG2D receptor on NK cells, CD8^+^, and γδ T cells, consequently reducing the cytotoxicity of these cells (Hedlund et al. 2009; Mincheva-Nilsson et al. 2006). Furthermore, clusters of FasL and TRAIL were identified on placental exosomes able to trigger apoptosis in T cells (Stenqvist et al. 2013). MSCs are another source of tolerogenic EVs. It has been reported that the regenerative effects in tissue injury exerted by MSCs are mediated in part by EVs and this includes an immunosuppressive component consisting of both RNA and proteins (Arslan et al. 2013; Burrello et al. 2016; Cantaluppi et al. 2012).

The immunosuppressive effects of donor-derived exosomes were even used to prolong graft survival after transplantation. Heart allograft survival in MHC-mismatched rats could be prolonged by injection of exosomes derived from donor bone marrow DCs before transplantation (Pêche et al. 2006). In addition, in a mouse model, exosomes isolated from immature DCs in combination with immunosuppressive drugs improved cardiac allograft survival (Li et al. 2012).

Immunoregulatory functions have been identified for several miRNAs transferred by EVs from non-transformed cells. Alexander et al. (2015) reported that exosomes can modulate the response to endotoxin-induced inflammation by transferring miRNA to antigen presenting cells. Two miRNAs that regulate inflammation, miR-146a and miR-155, were released from DCs within EVs and were taken up by recipient DCs. Injection of miR-146a-containing exosomes into mice inhibited the inflammatory response to endotoxin. A role of miR-146a in modulation of adaptive immunity was also suggested by Curtale et al. (2010). Up-regulation of miR-146a in T cells after stimulation of the TCR resulted in an anti-apoptotic signal counteracting activation-induced cell death. In exosomes derived from Foxp3^+^ Treg cells, let-7d was found to suppress Th1 cell proliferation and IFN-γ secretion (Okoye et al. 2014). A soluble T-cell suppressor factor recognized earlier to mediate antigen-specific inhibition of contact sensitivity was identified as miR-150. This miRNA was transported by exosomes derived from suppressor T cells (Bryniarski et al. 2013; Ptak et al. 2015) and the suppressive effect was dependent on the presence of macrophages (Nazimek et al. 2015).

As it is the case with all other mechanisms that support tumor growth, survival, and progression, also immunosuppression is not specific for cancer, but is abused during the disease to escape the immune surveillance program of the host.

## Conclusion

Tumors are heterogeneous, and different cells within the tumor may use different immune-escape mechanisms, such as apoptosis induction, impaired antigen presentation, or secretion of immunosuppressive factors. Moreover, multiple mechanisms may develop in a single tumor cell. Therefore, it is questionable whether a single, predominant immune-escape mechanism can be identified in a tumor. Exosomes participate in all kinds of mechanisms by which cancer evades immune surveillance and takes control over the immune system. Several aspects of the in vivo activity of exosomes are still unknown, especially how far they spread from the site of secretion and what quantities are secreted and captured by target cells. However, it is clear that cancer-derived exosomes are able to induce alterations of immune cell functions and a deeper insight into the cellular and molecular mechanisms underlying tumor immune escape using exosomes may finally lead to novel therapeutic approaches for the benefit of cancer patients.
